# Relationship between Undernutrition and Periodontal Diseases among a Sample of Yemeni Population: A Cross-Sectional Study

**DOI:** 10.1155/2022/7863531

**Published:** 2022-02-28

**Authors:** Milad Al-Kalisi, Manal Al-Hajri, Sarah Al-Rai

**Affiliations:** ^1^Oral Medicine, Oral Diagnosis and Periodontology Department, Faculty of Dentistry, Sana'a University, Sana'a, Yemen; ^2^Conservative and Preventive Department, Faculty of Dentistry, Saba University, Sana'a, Yemen

## Abstract

Undernutrition is an inadequate supply of energy and nutrients. Periodontal diseases (PDs) are defined as a broad form of chronic inflammatory disease of the gingiva, bone, and ligaments supporting the teeth. This study aimed to reveal the relationship between undernutrition, using body mass index (BMI) and serum albumin level (Alb), and PDs in a sample of Yemeni population. A cross-sectional study was conducted at dental teaching clinics at the Faculty of Dentistry, Sana'a University. Of 1920 patients who attended clinics, only 229 matched the study criteria. Oral examination was performed to assess the periodontal clinical parameter measurements. BMI and Alb were measured. Participants of both genders were involved, with a slight increase in males (*n* = 134, 58.5%), and most of the study sample was in the age group of 18–35 years (*n* = 209, 91.3%). Regarding habits, only 18.2% (*n* = 43) of patients were smokers and about half of the participants (*n* = 136, 59.4%) were khat chewers. Most cases had mild undernutrition according to BMI (*n* = 139, 60.7%) and normal Alb level (*n* = 213, 93%). Regarding the periodontal diagnosis, most of the participants were diagnosed with gingivitis (*n* = 186, 81.2%). BMI and albumin level were nonsignificantly associated with PDs. PDs were statistically significant with the participant's age, gender, level of education, and smoking (*P* > 0.05). However, BMI, khat chewing, and albumin level were nonsignificant factors of periodontal diseases among Yemeni participants (*P* > 0.05). In both genders, variables such as age of the patients, smoking, khat chewing, and PDs were nonsignificantly associated with BMI. This study showed that the majority of the participants had been diagnosed with gingivitis, but there was not an association between PDs and undernutrition. This paper is presented on research square URL // https://www.researchsquare.com/article/rs-429796/v1 with DOI 10.21203/rs.3.rs-429796/v1.

## 1. Background

Human nutritional status is a prerequisite for maintaining general health, including host recovery from different diseases. Undernutrition can be defined as a nutritional state resulting from a truly negative nutrient balance leading to a loss of body cell mass, including peripheral tissues (skeletal muscle, skin, and adipose tissue), whereas malnutrition is often considered to consist of a combination of undernutrition and inflammatory activity. Malnutrition can cause an alteration in the body composition, function, and clinical outcomes [1].

Periodontal diseases (PDs), including both gingivitis and periodontitis, are diseases induced by plaque and mainly affected by the immune and inflammatory responses, causing the breakdown of tooth tissues [2]. Plaque is the main cause of PDs. People with poor oral hygiene and plaque deposition showed a two-to-five-fold risk of periodontitis than people with good oral hygiene [3].

There are multiple risk factors that play important roles in an individual's response to periodontal infection [4, 5]. These risk factors can be classified as environmental, behavioral, or biological factors. Although the presence of these factors increases the rate of disease occurrence, their presence does not necessarily cause the disease. Risk factors are of two types: modifiable and nonmodifiable. The common modifiable factors are smoking [6, 7], diabetes mellitus [8–12], microbiome [13, 14], obesity [15, 16], tobacco, betel nut chewing [17, 18], and nutrition [19]. However, nonmodifiable factors may include genetic factors [20], ageing [21], gender [22, 23], and socioeconomic status (SES) [24].

Although oral microorganisms are responsible for the pathogenesis of PDs [25, 26], nutritional status can affect the balance between oral microorganisms and the host response which is a trigger of PD commencement and progression [26–28]. Undernutrition, specifically protein calorie, showed a reduction in immune host resistance especially the cellular immunity that causes an impairment in infection resistance [29].

Body mass index (BMI) is the most common anthropometric method that is used as an indicator to assess the nutritional status in nutritional and epidemiological studies with or without other anthropometric methods to assess patients at nutritional risk [30]. According to the World Health Organization (WHO), BMI interpretation is as follows: underweight individual is someone who has a BMI of <18.5 kg/m^3^, while a healthy individual has a BMI ranged between 18.5 and 24.9 kg/m^3^. Moreover, an overweight individual has a BMI ranged between 25 and 29.9 kg/m^3^, while an obese individual has a BMI of >30 kg/m^3^ [31]. Underweight can be classified as follows: mild undernutrition ranges from 17 to 18.5 kg/m^3^, moderate undernutrition ranges from 16 to 16.9 kg/m^3^, and severe underweight <16 kg/m^3^ [32]. Moreover, serum albumin level (Alb) can be used for identifying the inflammatory response and participating in the diagnosis of nutrient deficiency [33]. According to recent studies, biomarkers can predict the presence of periodontal diseases. According to Zhang et al. (2021), the combination of IL-1*β*, ICTP, and Pg can be used to distinguish stage III periodontitis subjects from healthy subjects and gingivitis subjects [34]. Also, the combination of IL-1*β* and MMP-8 can be used to differentiate between gingivitis patients and healthy subjects [34]. Another study showed that periodontitis patients had higher serum and salivary NLRP3 concentrations in comparison to healthy controls, which means that periodontitis could be a significant predictor of both serum and salivary NLRP3 concentrations [35]. In addition, another study demonstrated that periodontitis patients presented significantly higher salivary IL-6 levels compared with healthy patients [36].

According to the World Food Program (WFP), a recent survey showed that almost one-third of families have gaps in their diets and hardly ever consume foods like pulses, vegetables, fruit, dairy products, or meat. Malnutrition rates among women and children in Yemen remain among the highest in the world, with 1.2 million pregnant or breastfeeding women and 2.3 million children under 5 requiring treatment for acute malnutrition [37]. To our knowledge, there is no study that shows the relationship between periodontal disease and undernutrition. For this reason, this study aimed to provide current evidence on the prevalence of periodontal disease among undernourished participants and analyze the association between PDs and undernutrition, using body mass index (BMI) and serum albumin level (Alb) in a sample of Yemeni population.

## 2. Materials and Methods

This descriptive cross-sectional study was designed following STROBE guidelines and conducted in adherence to the Declaration of Helsinki. Ethical approval was obtained from the Human Ethics Committee of the Medical Faculty and Health Sciences of Sana'a University, Yemen. This study was conducted during the period from October 2018 to November 2019. Of 1290 patients who attended the postgraduate dental teaching clinics at the Faculty of Dentistry, Sana'a University, Yemen, only 229 patients matched the inclusion criteria. The inclusion criteria were determined as follows: undernourished patients with a BMI of <18.5 kg/m^3^ (mild undernutrition 17–18.5 kg/m^3^, moderate undernutrition 16–16.9 kg/m^3^, and severe underweight <16 kg/m^3^), both genders, age groups from 18 to 45 years, and free of systemic diseases. Exclusion criteria were as follows: people who has healthy weight or overweight or obesity, people who are older than 45 years or younger than 18 years, people who had systemic diseases, or people who were pregnant or lactating women. Written informed consent was collected from participants.

Sociodemographic data were collected through interviewing participants including age, gender, occupation, educational level, teeth cleaning frequency, smoking, and khat chewing. An oral examination was performed on the dental chair using sterile dental mirrors and Williams' probes by a single calibrated examiner (MA). Assessment of periodontal clinical parameter measurements was done including plaque index (PI), gingival index (GI), gingival recession (GR), probing pocket depth (PD), and clinical attachment loss (CAL). Kappa scores higher than 0.9 were attained for intraexaminer calibration exercises for identifying periodontal clinical parameters.

The undernourished patients' weights were measured in kilograms using a mechanical scale while participants wore light clothes and were without shoes. Moreover, the height of the participants was measured in centimeters using a measuring tape while a hard ruler was positioned horizontally over the head of the participant to ensure a stable base. BMI was calculated using the following formula: BMI = weight (kg)/height (m^2^). Blood samples were taken from each participant by a laboratory technician on the day of evaluation; samples were placed in a special container and sent to the laboratory to measure Alb level. Standard Alb ranges from 3.5 to 5.5 grams per deciliter (g/dl).

Data analysis was undertaken using the Statistical Package for Social Science (SPSS) (version 23.0). Several statistical tests were used as descriptive statistics using frequencies and percentages to present the sociodemographic data, habits, and other diagnostic variables such as IBM, albumin level, and periodontal diseases. Categorical variables were assessed using the Chi-square test. Furthermore, Fisher's exact test was used when the assumptions of the Chi-square test could not be met. An ordinal logistic regression model was used to identify the predictors for periodontal disease in the undernutrition young adult participants by finding the association between periodontal diseases and IBM, albumin level, age, gender, level of education, khat chewing, and smoking. A *P* value of less than 0.05 was considered statistically significant.

## 3. Results

A convincing sample of undernourished participants (*n* = 229) were enrolled from the dental teaching clinics at the Faculty of Dentistry, Sana'a University, between October 2018 and November 2019. Participants of both genders were involved in the study, with a slight increase in the number of males (*n* = 134, 58.5%), and most of the study sample was in the age group of 18–35 years (*n* = 209, 91.3%). Regarding habits, only 18.2% (*n* = 43) of patients were smokers and about half of the participants (*n* = 136, 59.4%) were khat chewers. Demographic data of the study subjects are shown in Table 1.

The presented study showed that most cases had mild undernutrition according to BMI (*n* = 139, 60.7%) and normal Alb level (*n* = 213, 93%). Regarding the periodontal diagnosis, most of the participants were diagnosed with gingivitis (*n* = 186, 81.2%), as shown in Table 2.

The bivariate results of the ordinal logistic regression showed that PDs were statistically significant with the participant's age, gender, level of education, and smoking (*P* ≤ 0.05). However, BMI, khat chewing, and albumin level were nonsignificant factors of periodontal diseases among Yemeni participants (*P* > 0.05), as shown in Table 3.

In both genders, variables such as age of the patients, smoking, khat chewing, and PDs were nonsignificantly associated with BMI, as presented in Table 4.

## 4. Discussion

This study aimed to assess the prevalence of periodontal disease among undernourished participants and analyze the association between PDs and undernutrition using body mass index (BMI) and serum albumin level (Alb) in a sample of the Yemeni population. The presented study showed that most of the cases had mild undernutrition according to BMI (*n* = 139, 60.7%) and normal Alb level (*n* = 213, 93%). The results showed that there was a nonstatistically significant association between PDs and undernutrition participants (*P* ≤ 0.05). Gingivitis was diagnosed in (81.2%) (*n* = 186) of study participants.

BMI is the most common noninvasive test to assess malnutrition [38]. Moreover, Alb is a well-known marker of nutritional status [39]. The use of biomarkers showed valuable results in many recent studies that showed a relation between the level of biomarkers and the diagnosis of periodontal diseases [33–36]. Low BMI and Alb in both genders were not significantly associated with PDs. Severe undernutrition has massive effects on the immune system, which plays a role in the progression of immunodeficiency [40], and the pathogenesis of periodontitis is significantly related to the host response in association with microbial factors [29, 41–43]. Cytokines are chemical mediators of inflammatory response that are influenced by nutritional status [44], so people with severe undernutrition are more susceptible to many microbial opportunistic infections [45]. In addition, the literature showed a positive correlation between hypoalbuminemia and periodontitis [46]. However, hypoalbuminemia requires a more destructive inflammation and severe undernutrition to occur [47]. Therefore, this may explain the nonsignificant relation between low BMI and Alb and PDs.

The presented study showed that most of the participants were between late adolescence and early adulthood with a slight increase in the number of males that have PDs, which was similar to that by Degarage et al., 2015 [48]. This can be explained by the neglectful behavior and the inability of males to take care of themselves, especially those who are studying away from their families and cannot cook food. Another explanation is poor oral hygiene. This can be due to the masculine behavior of thinking that oral hygiene is not connected to men's strength [49]. Regarding the occupation, most of the participants were students (60.3%) and most of them had an education level of bachelor's degree (49.8%). In this study, age, gender, education level, and smoking were significantly associated with PDs (*P* ≤ 0.05). Smoking is considered as one of the most significant lifestyle factors that is associated or linked to PDs and considered as a detrimental factor that influences the occurrence and progression of periodontitis [50, 51]. There is a high prevalence of khat chewing in Yemen (43.27%) [52]. Khat chewing habit is usually associated with the development of other habits like cigarette smoking [53, 54]. Khat chewing, which is a common practice among high school, college, and university students, is considered as a mild stimulant that promotes energy during working or studying [55]. Khat chewing can raise the concentration and energy levels at the beginning, and it can cause obvious CNS symptoms such as loss of appetite (anorexia) and may be associated with the mixed effects of the central amphetamine-like delaying of gastric emptying and insomnia that leads to waking up late in the morning and reduced activity performance caused by the central release of noradrenergic neurotransmitters [56–58]. Moreover, cathinone promotes or elevates the sympathomimetic activity that results in a late discharge of food from the stomach [59]. This may explain why khat chewing can lead to malnutrition. Most of the study sample was in the age group of 18–35 years, while chronic periodontitis is presented in the older age [60] and people of age 40 and above, who were four times more likely to have periodontitis than younger age groups [61].

The limitation of this study was the difficulty in convincing patients to participate in the study due to a lack of education. A further study can be done in the future with an increase in sample number.

## 5. Conclusions

Within the limitations of this study, most of the participants were diagnosed with gingivitis. Undernutrition is one of the health problems in the Yemen society. However, after using BMI and measuring albumin level, it was found that there was no association between PDs and undernutrition.

## Figures and Tables

**Table 1:** Sociodemographic characteristics of the study sample.

Variables		Frequency	%
Age	18–35	209	91.3
	35–45	20	8.7

Gender	Male	134	58.5
	Female	95	41.5

Occupation	Student	138	60.3
	House wife	32	14.0
	Retired	0	0.0
	Farmer	0	0.0
	Teacher	1	0.4
	Doctor	0	0.0
	Merchant	0	0.0
	Livestock breeder	0	0.0
	Craftsman	2	0.9
	Others	41	17.9
	Cannot find a job	15	6.6

Education level	Not educated	45	19.7
	Elementary	13	5.7
	Secondary	50	21.8
	Diploma	7	3.1
	Bachelor	114	49.8
	Master	0	0.0

Smoking	No	186	81.2
	Yes	43	18.8

Khat chewing	No	93	40.6
	Yes	136	59.4

**Table 2:** BMI, albumin level, and periodontal diagnosis of the study sample.

Variables		Frequency	%
BMI	Mild underweight	139	60.7
	Moderate underweight	59	25.8
	Severe underweight	31	13.5

Albumin level	Normal	213	93.0
	Low	16	7.0

Diagnosis	Healthy	9	3.9
	Gingivitis	186	81.2
	Chronic periodontitis	32	14.0
	Aggressive periodontitis	2	0.9

**Table 3:** Association between periodontal diseases and age, gender, level of education, smoking, khat chewing, BMI, and albumin level.

The variables		Estimate	Std. error	Wald	Df	Sig.	95% confidence interval	
							Lower bound	Upper bound
Dependent variable	Gingivitis	−3.80	1.41	7.24	1	0.01	−6.56	−1.03
	Chronic periodontitis	2.30	1.32	3.04	1	0.08	−0.29	4.89
	Aggressive periodontitis	5.73	1.55	13.76	1	0.00	2.70	8.76

Independent variables	Age	1.41	0.56	6.30	1	0.01	0.31	2.50
	Gender	−1.13	0.46	6.09	1	0.01	−2.03	−0.23
	Level of education	−0.36	0.13	7.56	1	0.01	−0.61	−0.10
	Smoking	1.11	0.49	5.18	1	0.02	0.15	2.06
	Khat chewing	0.41	0.51	0.64	1	0.42	−0.59	1.40
	Albumin level	0.07	0.57	0.02	1	0.90	−1.04	1.18
	BMI	0.30	0.25	1.48	1	0.22	−0.18	0.79

**Table 4:** Distribution of body mass index with age, smoking, khat chewing, and PDs categorized by gender.

Variables			BMI						*P* value
			Mild		Moderate		Severe		
			*N*	%	*N*	%	*N*	%	
Male	Age	18–35	79	90.8	28	93.3	15	88.2	0.814
		35–50	8	9.2	2	6.7	2	11.8	
	Smoking	No	71	81.6	24	80.0	11	64.7	0.303
		Yes	16	18.4	6	20.0	6	35.3	
	Khat chewing	No	19	21.8	5	16.7	3	17.6	0.855
		Yes	68	78.2	25	83.3	14	82.4	
	Periodontal disease diagnosis	Healthy	0	0.0	0	0.0	0	0.0	0.205
		Gingivitis	74	85.1	26	86.7	11	64.7	
		Chronic periodontitis	11	12.6	4	13.3	6	35.3	
		Aggressive periodontitis	2	2.3	0	0.0	0	0.0	

Female	Age	18–35	50	96.2	26	89.7	11	78.6	0.062
		35–50	2	3.8	3	10.3	3	21.4	
	Smoking	No	44	84.6	25	86.2	11	78.6	0.854
		Yes	8	15.4	4	13.8	3	21.4	
	Khat chewing	No	36	69.2	21	72.4	9	64.3	0.824
		Yes	16	30.8	8	27.6	5	35.7	
	Periodontal disease diagnosis	Healthy	5	9.6	3	10.3	1	7.1	0.327
		Gingivitis	43	82.7	23	79.3	9	64.3	
		Chronic periodontitis	4	7.7	3	10.3	4	28.6	
		Aggressive periodontitis	0	0.0	0	0.0	0	0.0	

Chi-square test and Fisher's exact test were used.

## Data Availability

Data can be made accessible to the interested researchers by the corresponding author on reasonable request.
